# 3,7-Dichloro­quinoline-8-carboxylic acid

**DOI:** 10.1107/S1600536808026238

**Published:** 2008-08-20

**Authors:** Xin-Hong Guo

**Affiliations:** a Huaiyin Teachers College, 111 West Changjiang Road, Huaian 223300, Jiangsu, People’s Republic of China

## Abstract

The title compound (trade name: quinclorac), C_10_H_5_Cl_2_NO_2_, was crystallized from a dimethyl sulfoxide solution. Quinclorac mol­ecules are packed mainly *via* π–π stacking inter­actions between neighbouring heterocycles (interplanar distance: 3.31 Å) and *via* O—H⋯N hydrogen bonding.

## Related literature

For the use of 3,7-dichloro­quinoline-8-carboxylic acid as a herbicide, see: Nuria *et al.* (1997 ▶); Pornprom *et al.* (2006 ▶); Sunohara & Matsumoto (2004 ▶); Tresch & Grossmann (2002 ▶). For related complexes, see: Li *et al.* (2008 ▶); Turel *et al.* (2004 ▶); Zhang *et al.* (2007 ▶).
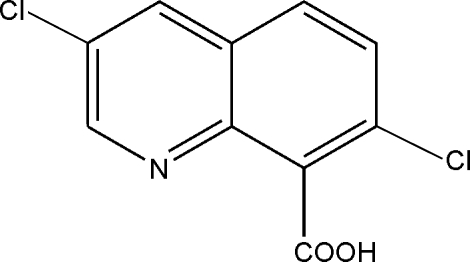


## Experimental

### 

#### Crystal data

C_10_H_5_Cl_2_NO_2_
*M* *_r_* = 242.05
Triclinic, 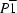

*a* = 7.5002 (12) Å
*b* = 8.4016 (14) Å
*c* = 8.732 (3) Å
α = 102.529 (6)°
β = 93.439 (6)°
γ = 116.479 (4)°
*V* = 472.98 (17) Å^3^
*Z* = 2
Mo *K*α radiation
μ = 0.66 mm^−1^
*T* = 173 (2) K
0.26 × 0.22 × 0.20 mm

#### Data collection

Bruker SMART APEXII diffractometer
Absorption correction: multi-scan (*SADABS*; Bruker, 1999 ▶) *T*_min_ = 0.84, *T*_max_ = 0.88
5948 measured reflections
1834 independent reflections
1102 reflections with *I* > 2σ(*I*)
*R*_int_ = 0.067

#### Refinement

*R*[*F* ^2^ > 2σ(*F* ^2^)] = 0.063
*wR*(*F* ^2^) = 0.140
*S* = 1.01
1834 reflections
139 parameters
H atoms treated by a mixture of independent and constrained refinement
Δρ_max_ = 0.30 e Å^−3^
Δρ_min_ = −0.43 e Å^−3^

### 

Data collection: *APEX2* (Bruker, 2004 ▶); cell refinement: *SAINT* (Bruker, 2004 ▶); data reduction: *SAINT*; program(s) used to solve structure: *SHELXS97* (Sheldrick, 2008 ▶); program(s) used to refine structure: *SHELXL97* (Sheldrick, 2008 ▶); molecular graphics: *ORTEP-3 for Windows* (Farrugia, 1997 ▶); software used to prepare material for publication: *SHELXL97* and *PLATON* (Spek, 2003 ▶).

## Supplementary Material

Crystal structure: contains datablocks global, I. DOI: 10.1107/S1600536808026238/zl2136sup1.cif

Structure factors: contains datablocks I. DOI: 10.1107/S1600536808026238/zl2136Isup2.hkl

Additional supplementary materials:  crystallographic information; 3D view; checkCIF report

## Figures and Tables

**Table 1:** Hydrogen-bond geometry (Å, °)

*D*—H⋯*A*	*D*—H	H⋯*A*	*D*⋯*A*	*D*—H⋯*A*
O1—H1*A*⋯N1^i^	0.84 (5)	1.91 (5)	2.753 (4)	173 (4)

Symmetry code: (i) 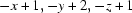.

**Fig. 1.**
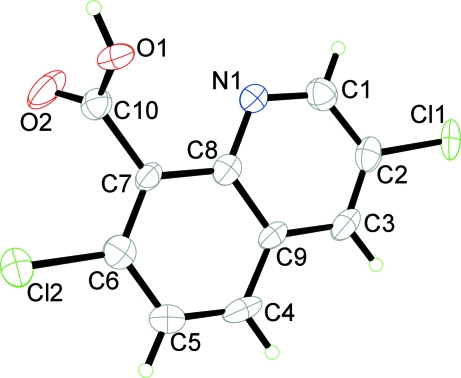
The asymmetric unit of quinclorac with atom labels and 50% probability displacement ellipsoids for non-H atoms.

**Fig. 2.**
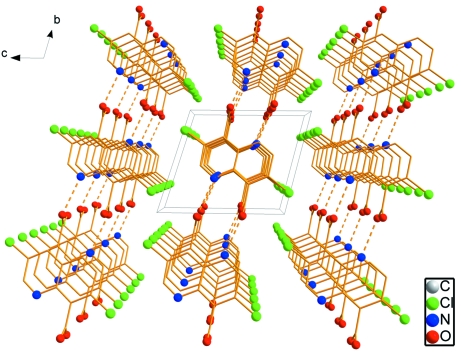
Packing diagram of quinclorac showing the π-π stacks along the a direction. Intermolecular H-bonding is indicated via dashed lines.

## supplementary crystallographic information

### Comment

Quinclorac (3,7-dichloroquinoline-8-carboxylic acid) is one of the most effective herbicides (Nuria *et al.*,  1997; Pornprom *et al.*,  2006; Sunohara & Matsumoto, 2004; Tresch & Grossmann, 2002), and is widely used in agriculture. In addition, as a quinolinecarboxylate derivate, quinclorac could chelate metal ions, forming corresponding complexes (Li *et al.*, 2008; Turel *et al.*,  2004; Zhang *et al.*, 2007). As an extension of these studies, we report herein on the structure of quinclorac.

A quinclorac molecule, which is the asymmetric unit of the structure, is shown in Fig. 1. All the bond distances and bond angles of quinclorac are normal and call for no further comment. Two types of intermolecular interations are easily found in the structure of quinclorac (Fig. 2). There exists a π-π interaction between adjacent quinin cycles with an inversion center located halfway between the aromatic rings, thus forming stacks along the *a* direction. Quinclorac molecules of adjacent chains are joined through H-bonding of O1—H1···N1^i^ (symmetry code: (i) 1 - *x*, 2 - *y*, 1 - *z*) (Table 1) into a triclinic supramolecular architecture (Fig. 2).

### Experimental

Quinclorac was obtained from a commercial source and used directly without further purification. Quinclorac (0.5 mmol, 0.121 g) was dissolved in 10 mL DMSO. After ether vapor slowly diffused into the solution at room temperature for several days, colorless prismlike crystals suitable for crystallographic research were obtained.

### Refinement

All the non-hydrogen atoms were located from the Fourier maps, and were refined anisotropically. The hydroxyl hydrogen, H1A, was found from the Fourier difference maps and refined isotropically with a fixed O—H bond length. All other H atoms were positioned geometrically. All isotropic vibration parameters of hydrogen atoms were related to the atoms which they are bonded to with *U*_iso_(H) = 1.2 *U*_eq_(C,O).

### Crystal data

**Table:**

C_10_H_5_Cl_2_NO_2_	*Z* = 2
*M**_r_* = 242.05	*F*_000_ = 244
Triclinic, *P*1	*D*_x_ = 1.700 Mg m^−^^3^
Hall symbol: -P 1	Mo *K*α radiation λ = 0.71073 Å
*a* = 7.5002 (12) Å	Cell parameters from 958 reflections
*b* = 8.4016 (14) Å	θ = 2.1–25.5º
*c* = 8.732 (3) Å	µ = 0.66 mm^−^^1^
α = 102.529 (6)º	*T* = 173 (2) K
β = 93.439 (6)º	Prismlike, colorless
γ = 116.479 (4)º	0.26 × 0.22 × 0.20 mm
*V* = 472.98 (17) Å^3^	

### Data collection

**Table:**

Bruker SMART APEXII diffractometer	1834 independent reflections
Radiation source: fine-focus sealed tube	1102 reflections with *I* > 2σ(*I*)
Monochromator: graphite	*R*_int_ = 0.067
*T* = 173(2) K	θ_max_ = 26.0º
ω scans	θ_min_ = 2.4º
Absorption correction: multi-scan(SADABS; Bruker, 1999)	*h* = −9→9
*T*_min_ = 0.84, *T*_max_ = 0.88	*k* = −8→10
5948 measured reflections	*l* = −10→10

### Refinement

**Table:**

Refinement on *F*^2^	Secondary atom site location: difference Fourier map
Least-squares matrix: full	Hydrogen site location: inferred from neighbouring sites
*R*[*F*^2^ > 2σ(*F*^2^)] = 0.063	H atoms treated by a mixture of independent and constrained refinement
*wR*(*F*^2^) = 0.140	*w* = 1/[σ^2^(*F*_o_^2^) + (0.062*P*)^2^] where *P* = (*F*_o_^2^ + 2*F*_c_^2^)/3
*S* = 1.01	(Δ/σ)_max_ < 0.001
1834 reflections	Δρ_max_ = 0.30 e Å^−^^3^
139 parameters	Δρ_min_ = −0.43 e Å^−^^3^
Primary atom site location: structure-invariant direct methods	Extinction correction: none

### Special details

**Table:**

Geometry. All esds (except the esd in the dihedral angle between two l.s. planes) are estimated using the full covariance matrix. The cell esds are taken into account individually in the estimation of esds in distances, angles and torsion angles; correlations between esds in cell parameters are only used when they are defined by crystal symmetry. An approximate (isotropic) treatment of cell esds is used for estimating esds involving l.s. planes.
Refinement. Refinement of F^2^ against ALL reflections. The weighted R-factor wR and goodness of fit S are based on F^2^, conventional R-factors R are based on F, with F set to zero for negative F^2^. The threshold expression of F^2^ > 2sigma(F^2^) is used only for calculating R-factors(gt) etc. and is not relevant to the choice of reflections for refinement. R-factors based on F^2^ are statistically about twice as large as those based on F, and R- factors based on ALL data will be even larger.

### Fractional atomic coordinates and isotropic or equivalent isotropic displacement parameters (Å^2^)

**Table:**

	*x*	*y*	*z*	*U*_iso_*/*U*_eq_	
C1	0.3476 (6)	0.5471 (6)	0.2465 (5)	0.0354 (10)	
H1	0.3861	0.5957	0.1583	0.043*	
C2	0.2932 (6)	0.3616 (6)	0.2276 (5)	0.0324 (9)	
C3	0.2343 (6)	0.2876 (6)	0.3515 (5)	0.0337 (10)	
H3	0.1963	0.1613	0.3413	0.040*	
C4	0.1686 (6)	0.3342 (6)	0.6269 (5)	0.0369 (10)	
H4	0.1286	0.2086	0.6220	0.044*	
C5	0.1655 (7)	0.4475 (6)	0.7605 (5)	0.0363 (10)	
H5	0.1215	0.4011	0.8489	0.044*	
C6	0.2272 (6)	0.6342 (6)	0.7699 (5)	0.0308 (9)	
C7	0.2918 (6)	0.7078 (5)	0.6455 (5)	0.0253 (8)	
C8	0.2910 (5)	0.5882 (5)	0.5036 (5)	0.0261 (8)	
C9	0.2304 (6)	0.4002 (5)	0.4943 (5)	0.0261 (8)	
C10	0.3645 (6)	0.9105 (5)	0.6610 (4)	0.0293 (9)	
Cl1	0.29545 (15)	0.22889 (15)	0.04666 (12)	0.0385 (3)	
Cl2	0.23077 (17)	0.77619 (16)	0.94953 (12)	0.0422 (3)	
N1	0.3496 (5)	0.6591 (4)	0.3774 (4)	0.0283 (7)	
O1	0.5586 (4)	0.9997 (4)	0.6659 (3)	0.0302 (6)	
H1A	0.597 (7)	1.106 (7)	0.652 (5)	0.036*	
O2	0.2510 (5)	0.9766 (4)	0.6634 (5)	0.0519 (9)	

### Atomic displacement parameters (Å^2^)

**Table:**

	*U*^11^	*U*^22^	*U*^33^	*U*^12^	*U*^13^	*U*^23^
C1	0.038 (2)	0.041 (3)	0.032 (2)	0.021 (2)	0.0079 (18)	0.012 (2)
C2	0.027 (2)	0.032 (2)	0.039 (2)	0.0174 (19)	0.0036 (17)	0.0030 (19)
C3	0.033 (2)	0.021 (2)	0.049 (2)	0.016 (2)	0.0051 (19)	0.008 (2)
C4	0.037 (2)	0.025 (2)	0.053 (3)	0.014 (2)	0.006 (2)	0.022 (2)
C5	0.043 (2)	0.035 (3)	0.036 (2)	0.019 (2)	0.0106 (19)	0.017 (2)
C6	0.0262 (19)	0.035 (2)	0.033 (2)	0.0165 (18)	0.0041 (16)	0.0098 (19)
C7	0.028 (2)	0.020 (2)	0.032 (2)	0.0138 (17)	0.0062 (16)	0.0073 (17)
C8	0.0180 (18)	0.024 (2)	0.034 (2)	0.0095 (16)	0.0014 (15)	0.0050 (17)
C9	0.0258 (18)	0.020 (2)	0.036 (2)	0.0126 (16)	0.0030 (16)	0.0091 (17)
C10	0.034 (2)	0.025 (2)	0.0246 (19)	0.0119 (18)	0.0010 (15)	0.0038 (18)
Cl1	0.0372 (6)	0.0432 (7)	0.0423 (6)	0.0303 (5)	0.0101 (5)	−0.0006 (5)
Cl2	0.0548 (7)	0.0433 (7)	0.0343 (6)	0.0280 (6)	0.0144 (5)	0.0089 (5)
N1	0.0318 (18)	0.0235 (18)	0.0295 (17)	0.0130 (15)	0.0066 (13)	0.0069 (15)
O1	0.0319 (16)	0.0189 (15)	0.0364 (16)	0.0071 (13)	0.0056 (12)	0.0120 (13)
O2	0.048 (2)	0.0257 (17)	0.092 (3)	0.0240 (16)	0.0204 (18)	0.0171 (18)

### Geometric parameters (Å, °)

**Table:**

C1—N1	1.308 (5)	C5—H5	0.9500
C1—C2	1.391 (6)	C6—C7	1.373 (6)
C1—H1	0.9500	C6—Cl2	1.743 (4)
C2—C3	1.362 (6)	C7—C8	1.414 (5)
C2—Cl1	1.731 (4)	C7—C10	1.510 (5)
C3—C9	1.403 (6)	C8—N1	1.369 (5)
C3—H3	0.9500	C8—C9	1.417 (5)
C4—C5	1.345 (6)	C10—O2	1.206 (5)
C4—C9	1.405 (5)	C10—O1	1.299 (5)
C4—H4	0.9500	O1—H1A	0.84 (5)
C5—C6	1.405 (6)		
			
N1—C1—C2	124.6 (4)	C7—C6—Cl2	119.9 (3)
N1—C1—H1	117.7	C5—C6—Cl2	118.0 (3)
C2—C1—H1	117.7	C6—C7—C8	117.8 (4)
C3—C2—C1	118.9 (4)	C6—C7—C10	121.0 (3)
C3—C2—Cl1	121.5 (3)	C8—C7—C10	121.2 (3)
C1—C2—Cl1	119.6 (3)	N1—C8—C7	118.2 (4)
C2—C3—C9	119.2 (4)	N1—C8—C9	121.6 (3)
C2—C3—H3	120.4	C7—C8—C9	120.2 (4)
C9—C3—H3	120.4	C3—C9—C4	122.9 (4)
C5—C4—C9	120.5 (4)	C3—C9—C8	118.0 (4)
C5—C4—H4	119.7	C4—C9—C8	119.1 (3)
C9—C4—H4	119.7	O2—C10—O1	125.4 (4)
C4—C5—C6	120.2 (4)	O2—C10—C7	122.5 (3)
C4—C5—H5	119.9	O1—C10—C7	112.1 (3)
C6—C5—H5	119.9	C1—N1—C8	117.7 (3)
C7—C6—C5	122.1 (4)	C10—O1—H1A	113 (3)

### Hydrogen-bond geometry (Å, °)

**Table:**

*D*—H···*A*	*D*—H	H···*A*	*D*···*A*	*D*—H···*A*
O1—H1A···N1^i^	0.84 (5)	1.91 (5)	2.753 (4)	173 (4)

Symmetry codes: (i) −*x*+1, −*y*+2, −*z*+1.
